# 

**Published:** 1907-11-02

**Authors:** 


### POST GRADUATE LECTURES IN LONDON NEXT WEEK.

The Post Graduate College, West London Hospital, Hammersmith, W.—At 5 p.m.—November 4, Dr. Saunders, Clinical; November 5, Dr. Low, Sleeping Sickness; November 6, Dr. Beddard, Practical Medicine; November 7, Mr. Bidwell, Clinical; November 8, Dr. Abraham, Cases of Skin Disease. Clinical demonstrations daily.

The London School of Clinical Medicine, Seamen's Hospital, Greenwich.—At 4 p.m.—November 4, Dr. St. Clair Thomson, Laryngeal Paralysis. At 3.30 p.m.—November 6, Mr. Cargill, Ophthalmic Diagnosis. Clinical demonstrations daily.

The Medical Graduates' College and Polyclinic, Chenies Street, W.C.—5.15 p.m.—November 4, Dr. F. J. Smith, Heart Disease without Murmurs; November 5, Dr. Nathan Raw, Human and Bovine Tuberculosis; November 6, Dr. C. R. Box, Bacterial Invasion of the Urinary Tract in Childhood; November 7, Dr. T. Grainger Stewart, The Diagnosis of Cerebellar Tumours. Clinical demonstrations and practical classes daily.

THE HOSPITAL *November* 2, 1907.

*Name* ..................................................

*Address* ..................................................

..................................................

This Coupon must accompany manuscript or contributions intended for The Hospital.

